# Chromosomal Speciation Revisited: Modes of Diversification in Australian Morabine Grasshoppers (*Vandiemenella*, *viatica* Species Group)

**DOI:** 10.3390/insects2010049

**Published:** 2011-03-18

**Authors:** Takeshi Kawakami, Roger K. Butlin, Steven J. B. Cooper

**Affiliations:** 1 Division of Biology, Kansas State University, Manhattan, Kansas 66506, USA; E-Mail: kawakami.t@gmail.com; 2 Animal and Plant Sciences, University of Sheffield, Sheffield S10 2TN, UK; E-Mail: r.k.butlin@sheffield.ac.uk; 3 Evolutionary Biology Unit, South Australian Museum, Adelaide, SA 5000, Australia; 4 Australian Centre for Evolutionary Biology and Biodiversity, The University of Adelaide, SA 5005, Australia

**Keywords:** chromosomal rearrangements, hybridization, mtDNA introgression, phylogeography, population genetics, selection, speciation

## Abstract

Chromosomal rearrangements can alter the rate and patterns of gene flow within or between species through a reduction in the fitness of chromosomal hybrids or by reducing recombination rates in rearranged areas of the genome. This concept, together with the observation that many species have structural variation in chromosomes, has led to the theory that the rearrangements may play a direct role in promoting speciation. Australian morabine grasshoppers (genus *Vandiemenella*, *viatica* species group) are an excellent model for studying the role of chromosomal rearrangement in speciation because they show extensive chromosomal variation, parapatric distribution patterns, and narrow hybrid zones at their boundaries. This species group stimulated development of one of the classic chromosomal speciation models, the stasipatric speciation model proposed by White in 1968. Our population genetic and phylogeographic analyses revealed extensive non-monophyly of chromosomal races along with historical and on-going gene introgression between them. These findings suggest that geographical isolation leading to the fixation of chromosomal variants in different geographic regions, followed by secondary contact, resulted in the present day parapatric distributions of chromosomal races. The significance of chromosomal rearrangements in the diversification of the *viatica* species group can be explored by comparing patterns of genetic differentiation between rearranged and co-linear parts of the genome.

## 1. Introduction

Speciation is the evolutionary process that leads to barriers to gene exchange between taxa, and understanding the factors that give rise to such barriers is a central theme of evolutionary biology [1]. Variation in the structure of chromosomes has long been considered to provide barriers to gene flow between hybridizing taxa with different karyotypes; however, whether such changes play a direct role in the process of speciation has been much debated [2–4]. Traditional chromosomal theories of speciation predict that structural chromosomal changes, such as inversions, fusions, and translocations, play a causative role in reproductive isolation through reducing fitness in heterokaryotypes (reviewed in [4,5]). Development of these models is based on the observation that natural and laboratory-bred chromosomal heterozygotes have abnormalities of meiosis, which impair the fertility of chromosomal hybrids. Although certain types of chromosomal rearrangements have the opportunity to be actively involved in speciation processes by reducing fertility of chromosomal heterozygotes (e.g., monobrachial centric fusions [6,7]), many of the traditional chromosomal speciation models suffer from critical theoretical problems. Specifically, if fitness reduction by chromosomal rearrangements is sufficiently high to reduce gene flow and promote speciation, then it may be difficult for these new rearrangements to become fixed in populations [5,8]. Because of these theoretical problems, others view chromosomal rearrangements as incidental by-products of speciation processes (e.g., [3]).

Recent theoretical studies, which have reinvigorated the chromosomal speciation field, have suggested alternative roles of chromosomal rearrangements in speciation: either by accelerating divergence between populations through the spread of locally adapted alleles or by protecting combinations of genes associated with reproductive isolation [5,9–11]. These new models (referred to as the suppressed-recombination models [12]) rely more on the reduction in recombination caused by chromosomal rearrangements than traditional theories, which were dependent on their fitness effects in hybrids. The suppressed-recombination models suggest that in the early stages of speciation, some parts of the genome involved in reproductive isolation or local adaptation will show restricted patterns of gene flow, while other parts of the genome not linked to ‘isolation or adaptation genes’ will show limited differentiation between hybridizing taxa. To date, only a limited number of empirical systems have been used to examine the role of chromosomal rearrangements under these new theories (e.g., sunflowers [13,14], monkeyflower [15], *Drosophila* [9,16,17], mosquitoes [18], shrews [19] and house mouse [20]).

**Figure 1:**
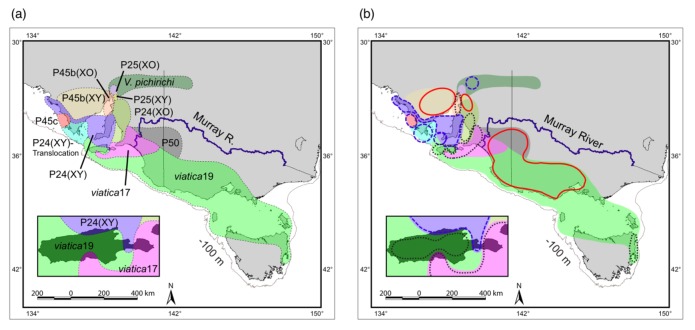
**(a)** Parapatric distribution of chromosomal races of the *viatica* species group in southeastern Australia proposed by White *et al.* [21,23]. An inset shows distribution of three races on Kangaroo Island. A 100 m isobath is indicated as a proxy of an ancient coastline at glacial maxima during the Pleistocene. Karyotypes of each race (♂)/(♀) are: *viatica*19, 2n = 19/20, XO/XX; *viatica*17, 2n = 17/18, XO/XX; P24(XO), 2n = 17/18, XO/XX; P24(XY), 2n = 16, XY/XX; P24(XY)-Translocation, 2n = 16, XY/XX; P25(XO), 2n = 19/20, XO/XX; P25(XY), 2n = 18, XY/XX; P45b(XO), 2n = 19/20, XO/XX; P45b(XY), 2n = 18, XY/XX; P50, 2n = 19/20, XO/XX; *V. pichirichi*, 2n = 19/20, XO/XX. **(b)** Thirteen genetic clusters resolved by the Bayesian clustering analysis using 35 allozyme loci, superimposed on a distribution map. Red circles with solid line indicate clusters shared among multiple chromosomal races. Four taxa [P24(XY), P24(XY)-Translocation, P45c, and *V. pichirichi*] comprise exclusive genetic clusters (blue circles with dashed line).

Australian morabine grasshoppers of the genus *Vandiemenella* (the *viatica* species group) provide an excellent study system to investigate potential roles of chromosomal rearrangements in speciation because they show extensive chromosomal variation, with 12 known chromosomal races/species [2,21]. These taxa have been discriminated by chromosomal rearrangements (fusions, fissions, translocations, or inversions), characters of the external genitalia, and morphometrics [21,22]. With only two exceptions, most taxa have parapatric distributions, in a mosaic pattern within South Australia (SA), and often form narrow contact zones at their boundaries (Figure 1a). White and colleagues carried out extensive studies of a number of the hybrid zones on Kangaroo Island and the mainland of SA, and controlled breeding studies of hybrids between a number of the chromosomal races, providing a background of data on the chromosomal variation and fitness of hybrids [21–27]. In contact zones, two different chromosomal taxa meet, hybridize, and produce at least some offspring of mixed ancestry, forming a smooth transition of chromosomal, morphological, and some heritable characters (each character transition is termed a ‘cline’). Largely based on these studies in the *viatica* species group, White argued that chromosomal changes play a causative role in speciation by leading to hybrid dysfunction or underdominance of heterokaryotypic individuals and proposed a classic chromosomal speciation model, called the ‘stasipatric speciation model’ [2,28]. Key features of this model include (i) chromosomal rearrangements produce barriers to gene flow between parental and daughter chromosome types due to meiotic abnormalities in chromosomal heterozygotes, and (ii) the spread of new chromosome types from their point of origin into the distribution of a parental chromosome type occurs without geographic isolation, leading to parapatric distributions of chromosomal races. However, because this model also suffers from the theoretical problems described earlier, this mechanism is no longer considered viable [1].

Despite the pioneering work of White and colleagues on the *viatica* species group over 40 years ago, we still know very little about whether chromosomal rearrangements play a critical role in the diversification within the group. How does chromosomal divergence correlate with genetic divergence? Are chromosomal variants in the group associated with barriers to gene flow? Is there any evidence for an allopatric phase during diversification of the group, or have chromosomal races diversified without geographic isolation as the stasipatric model predicts? To provide new insights into these questions, we have explored the population genetic structure and phylogeography of the group across their range in southeastern Australia and investigated the level of introgression of molecular genetic markers among the chromosomal taxa using a combination of allozymes, microsatellites, two nuclear DNA sequences (*Elongation Factor* 1α [*EF*-1α] and an anonymous nuclear marker *Mvia11*) and one mitochondrial cytochrome c oxidase subunit I (*COI*) sequence marker [29–33]. Our aim here is to review these recent molecular studies, bringing the results from hybrid zone, phylogeographical and population genetic analyses together, to reassess the potential role of chromosomal variation in promoting diversification of the *viatica* species group. We also highlight that this classic study system provides an unprecedented opportunity to explore the new chromosomal speciation theories.

## 2. Chromosomal and Genetic Divergence

One of the keys to understanding the potential roles of chromosomal rearrangements in the diversification of the *viatica* species group is to investigate how much genetic variation is associated with chromosomal variations. The stasipatric model *sensu stricto* predicts that a substantial portion of the total genetic variation is explained by the chromosomal variation because all the speciation/diversification events are accompanied by chromosomal mutations, which create strong barriers to gene flow between parental and daughter chromosomal races due to meiotic abnormalities in chromosomal heterozygotes [2,28]. However, if the contribution of other isolating mechanisms was greater, then the proportion of the genetic variation explained by the chromosomal variation should be lower. These general predictions were tested by applying population genetic and phylogeographic analyses to molecular and cytological data.

We first focus on three chromosomal races of the *viatica* species group on Kangaroo Island because the small island setting provides relatively simple conditions without potentially confounding effects of migrations from populations on the Australian mainland. There are two isolated populations of *viatica*17 (2n = 17/18, XO/XX), two isolated populations of P24(XY) (2n = 16, XY/XX), and one widely distributed population of *viatica*19 (2n = 19/20, XO/XX) (Figure 1a). Phylogenetic and population clustering analyses using two independent sets of nuclear markers (allozymes and *EF*-1α gene) resolved individuals from the three chromosomal races into three genetically distinct groups, regardless of their geographical distribution (Figure 1b) [30]. The only exceptions were individuals that were collected from the sites near the P24(XY)-*viatica*17 contact zone. These individuals had allozyme and *EF*-1α alleles typical of the alternative chromosomal races, suggesting ongoing gene introgression across the zone. Overall, strong genetic structure in nuclear markers with many loci fixed with race- specific alleles suggests substantial divergence with limited gene flow of the nuclear genome. An approximate estimate of the divergence time between these races is 1.0–3.7 million years ago based on a conventional molecular clock for allozymes [34] and an inferred substitution rate of the *EF*-1α exon region in *Papilio* butterflies [35].

When all the 12 chromosomal taxa distributed on mainland Australia, Tasmania, and Kangaroo Island were analyzed, the association between genetic and chromosomal variation was not as clear as that found in the Kangaroo Island populations. The Analyses of Molecular Variance (AMOVA) revealed that chromosomal variants explain about half of the total genetic variation in the three nuclear markers (40% in allozyme, 51% in anonymous *Mvia11* locus, and 65% in *EF*-1α locus), and the remaining half of the total genetic variation is explained by between populations within chromosomal taxa (29% in allozyme, 30% in anonymous *Mvia11* locus, and 26% in *EF*-1α locus) and within populations (31% in allozyme, 19% in anonymous *Mvia11* locus, and 9% in *EF*-1α locus). Phylogenetic analyses using these three nuclear markers show moderate differentiation among the chromosomal taxa; however, extensive non-monophyly is prevalent with numerous shared alleles among the taxa. Including taxa represented by single alleles, only five taxa formed monophyletic or nearly monophyletic groups for *EF*-1α, and one taxon formed a monophyletic group for *Mvia11*. The weak correspondence between molecular markers and chromosomal variants is further supported by allozyme clustering analyses implemented by STRUCTURE and BAPS. These Bayesian clustering analyses resolved 13 genetic clusters within the species group; only four chromosomal taxa formed exclusive genetic clusters, while the other seven taxa share clusters with another taxon (Figure 1b). In general, discordant genetic structure can result from incomplete lineage sorting of ancestral polymorphisms and/or historical and contemporary gene introgression [36]. Both mechanisms probably have played a part in the genetic differentiation of the *viatica* species group; however, hybrid zone analyses using a contact zone between P24(XY) and *viatica*17 races [29] suggest that ongoing gene introgression plays an important role in the genetic admixture between parapatric taxa (see the next section). Overall, while the *Vandiemenella* chromosomal taxa generally represent genetically distinct units in the nuclear genome, a substantial portion of the total genetic variation was not explained by the chromosomal variation.

Discordance between chromosomal variation and genetic variation is more pronounced in the mitochondrial genome than the nuclear genome. AMOVA revealed that only 15% of total genetic variation in the *COI* locus was explained by chromosomal variation [31]. Haplotype network analyses based on the statistical parsimony method (TCS, [37]) showed extensive sharing of haplotypes among multiple chromosomal taxa, while also showing the existence of multiple genetically distinct groups within taxa. Similar to the loci in the nuclear genome, both incomplete lineage sorting and gene introgression can be sources of the discordance between chromosomal and mitochondrial markers. However, hybrid zone analyses suggest weaker barriers to gene flow in the mitochondrial genome than the nuclear genome (see the next section), implying a substantial contribution of gene introgression in the formation of population genetic structure associated with the mitochondrial genome [29].

## 3. Evidence of Allopatry

Geographical modes of speciation/diversification within the *Vandiemenella viatica* species group have been contentious. White [2,28] hypothesized that the distribution of *Vandiemenella* was ‘essentially’ contiguous through the entire process of chromosomal diversification, and that allopatric processes played little or no role in the diversification within the group. This supposition further emphasizes that narrow hybrid zones between taxa have been maintained since the emergence of daughter chromosome types (*i.e.*, primary origin of contact zones). In contrast, Key [38] and Hewitt [39] argued that peripheral allopatric origins of daughter chromosomal variants are more likely, and contact zones between the chromosomal taxa were formed by secondary contact after vicariance. Recent phylogeographic studies highlight the substantial impact of the past climate changes on distribution and diversity of Australian biota [40–44]. Therefore it is possible that the past climate oscillations have affected chromosomal diversification and population genetic structure of the *viatica* species group. Specifically, it is important to test i) whether allopatric fragmentation has played a role in the diversification of the *viatica* species group, and ii) whether the origins of parapatric distributions of chromosomal races of the *viatica* species group were primary or secondary.

Phylogeographic and population genetic analyses using data from allozymes and two nuclear sequence markers support the involvement of allopatric fragmentation within the *viatica* species group [31]. For example, *viatica*19, which has the widest distribution among the *Vandiemenella* taxa, is composed of multiple, genetically divergent populations (Figure 1b). White [26,45] proposed that this race represents a single ancestral karyotypic lineage based on the comparative cytological studies within the subfamily Morabinae; however, the existence of highly divergent populations particularly at the periphery of the distribution including Tasmania and Kangaroo Island suggests a long-term persistence of *viatica*19 in geographically isolated populations. Similar genetic subdivision is also revealed within *viatica*17: one northwest of the Murray River including Kangaroo Island and the other southeast of the Murray River. In addition to the Murray River, active dune fields during recent glacial maxima in the Pleistocene [33] and the ancient Lake Bungunnia occupying the Western Murray Basin about 0.7–3.5 million years ago [46] may have provided a long-term physical barrier to gene flow in this chromosomal race, as found previously in a study of marsupials [47] and, more recently, in the reptile *Tiliqua rugosa* [48]. Furthermore, high levels of genetic and cytological diversity in the region towards the northwest of the distribution range may be attributable to repeated population subdivisions and range shifts due to sea level fluctuations, and appearance/disappearance of the gulfs and peninsulas through climatic oscillations in this region. These results highlight the clear correlation between boundaries of population subdivisions and biogeographic barriers, but the boundaries of these population subdivisions do not always correspond with parapatric boundaries defined by karyotypes.

Signatures of past demographic changes during putative allopatric fragmentation are revealed by population genetic analyses in the three chromosomal races on Kangaroo Island. Neutrality/demography tests (Tajima's D [49], Fu's *F*_s_ [50], *R*_2_ of Ramos-Onsins and Rozas [51]), mismatch distribution tests [52], and population growth parameter *g* in coalescent simulations [53,54] detected signals of population growth for all three races in the mitochondrial *COI* and nuclear *EF*-1α sequences [30]. Given the estimated divergence time of the three chromosomal races of >1.0 million years ago, these demographic changes may have coincided with glacial-interglacial cycles during the Pleistocene. Geological evidence suggests that northwesterly winds from the continental interior made the environment on Kangaroo Island more arid and less vegetated when the island was joined to mainland Australia during glacial maxima [46,55]. During this period, a series of contractions and expansions of this grasshopper's habitat may have created locally isolated populations and historical contact zones (e.g., in regions now located under water) [39], which allowed gene introgression between races and subsequent fixation of alleles from one chromosomal race in the range of a second chromosomal race (e.g., the fixation of *viatica*19 mtDNA in the range of *viatica*17 from south-coast Kangaroo Island [30]).

**Figure 2:**
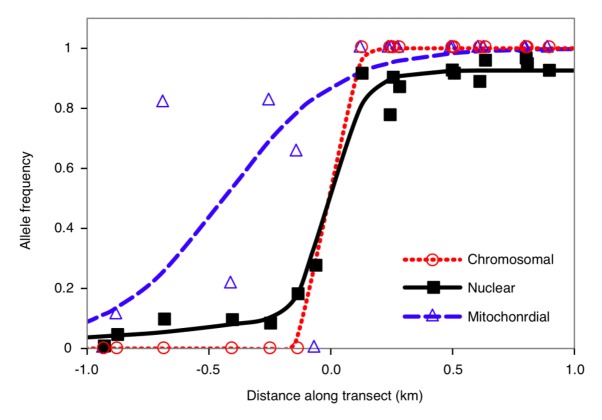
Estimated allele frequency clines of chromosomal (dotted red line), nuclear (solid black line) and mitochondrial markers (dashed blue line) across a hybrid zone between P24(XY) (to the left side) and *viatica*17 (to the right side) on Kangaroo Island based on maximum likelihood models. Best fit models are the sigmoid model for the chromosomal and mitochondrial markers and asymmetrical stepped model for the nuclear markers. Circles, squares, and triangles represent observed allele frequencies of chromosomal, nuclear (average of 10 autosomal loci), and mitochondrial markers, respectively. Distance is expressed relative to the center (= 0 km) of the average nuclear cline.

A secondary origin of the parapatric distribution is supported by the narrowness of the hybrid zones relative to the dispersal rate of grasshoppers and patterns of gene introgression across a hybrid zone between P24(XY) and *viatica*17 on Kangaroo Island [29]. Hybrid zone analyses using a maximum-likelihood approach gave an average estimate of cline width *w* = 310 m and dispersal parameter σ = 48.7 m/generation^1/2^ for 10 autosomal markers (Figure 2). If the contact zone between these two races had a primary origin > 1.0 million years ago, hybrid zone widths for putatively neutral markers should have been much wider than 310 m. In addition, widths and positions of clines for these ten nuclear markers were concordant and coincident with the chromosomal cline. Such concordant and coincident clines are thought to originate from secondary contact between the chromosomal races or strong linkage between nuclear and chromosomal markers. Although genomic locations of these nuclear markers are unknown, it is unlikely that all ten autosomal markers are located on the rearranged chromosomes (*i.e.*, X chromosome inversion and a fusion between the inverted X chromosome and an acrocentric autosome). In contrast to the nuclear markers, the mitochondrial *COI* marker showed a significantly wider cline (911 m) with center offset toward the P24(XY) side (409 m). Although there are several mechanisms that could result in the discordance between the mitochondrial and nuclear clines, such as asymmetrical hybridization, differences in fitness, differences in female mate preference and male aggression, hybrid zone movement toward the *viatica*17 side after secondary contact would be the most likely given the overall asymmetry of the clines in the nuclear markers. The results further suggest that reduction of nuclear gene flow may be associated with the chromosomal variation, or underlying genetic variation linked with chromosomal variation, whereas mtDNA gene flow appeared to be independent of this variation.

## 4. Conclusions

The *viatica* species group of Australian morabine grasshoppers provided an important study system in the development of early chromosomal speciation theory in the 1960s, but its mode of speciation/diversification has been the subject of considerable debate. A series of population genetic and phylogeographic studies [29–31] revealed that associations between genetic and chromosomal variation are limited to certain karyotypic lineages, and the extensive non-monophyly of chromosomal races indicates that diversification within the group was not always accompanied by chromosomal mutations. Introgressive hybridization between the chromosomal races appears to be involved in the formation of these discordant population patterns. Patterns of genetic differentiation between the chromosomal races show dynamic responses of the grasshoppers to past climatic fluctuations, leading to opportunities for long-term isolation and allopatric fixation of new chromosome variants and molecular mutations at many loci. Concordant and coincident clines for nuclear and chromosomal markers in the P24(XY)-*viatica*17 contact zone suggest that it formed by secondary contact and that barriers to gene flow in the nuclear genome are generally associated with the chromosomal variation. Although not statistically significant, the chromosomal variants (involving the X-chromosome and one of the autosomes) showed a narrower cline than any of the clines for nuclear markers, suggesting that selection is strongest for the chromosomal rearrangements.

Taken together, our phylogeographic, population genetic and hybrid zone analyses, suggest that different parts of the genome, in addition to the chromosomal variation, are under selective constraints. However, there are a number of limitations. First, we have no knowledge of where any of the markers map in the genome. It is possible that some are located on rearranged chromosomes, or act in combination with markers on the chromosomal rearrangements. Second, there was insufficient power in the analyses to provide statistical support for the chromosomal variants being under stronger selection than the other nuclear makers.

In order to discriminate between reproductive barriers caused only by genetic factors or in combination with chromosomal differences, further analyses are required to investigate differential gene flow using a large number of genetic markers within and outside the rearranged chromosome regions. Such analyses are particularly relevant for assessing the potential role of the suppressed- recombination models, where chromosomal rearrangements may facilitate diversification after secondary contact by reducing recombination and so maintaining linkage disequilibria between genes in a large block of the genome [5,9]. Such large blocks of the genome protected from recombination (often referred to as ‘genomic islands of differentiation’ [56]) would provide a strong genetic barrier if these genomic blocks have accumulated alleles that are incompatible with a foreign genetic background, or allele combinations favorable for the local environment, during an allopatric phase. Because these genomic islands tend to remain differentiated in the face of gene introgression, identification of such genomic islands is useful for tracking down individual genes that are responsible for reproductive isolation and/or differential environmental adaptation [57]. Comparative genome scan approaches have proven to be useful to identify such genomic islands with elevated genetic differentiation between closely related species [58,59].

One of the exciting features of the *viatica* species group is that various types and numbers of chromosomal rearrangements are involved, there are divergent populations that lack chromosomal differences, and a number of hybrid zones composed of different combinations of chromosomal races are available for study. It is expected that different pairs of chromosomal races, different levels of genetic divergence through different degrees of isolation in allopatry, and different ages of contact zones (e.g., possibly *viatica*19 and *viatica*17 contact zones on Kangaroo Island and the mainland Australia), may show different levels of genetic admixture between pairs of chromosomal races and, hence, have different sizes/numbers of genomic islands. We believe that the new molecular data in addition to the extensive chromosomal variation make the *viatica* species group an ideal model system for further exploring the potential role of chromosomes in speciation.
